# Gastric lavage may not be representative of total microplastic ingestion for a wild passerine bird

**DOI:** 10.1371/journal.pone.0334891

**Published:** 2025-10-23

**Authors:** R. Keith Andringa, Nicholas A. Bruni, Jennifer A. Smith, Heather L. Prestridge, Ryan Thornton, Jacquelyn K. Grace

**Affiliations:** 1 Ecology and Evolutionary Biology Interdisciplinary Program, Texas A&M University; 2 Department of Ecology and Conservation Biology, Texas A&M University; 3 Biodiversity Research and Teaching Collections, Department of Ecology and Conservation Biology, Texas A&M University; 4 Schubot Center for Avian Health, Department of Veterinary Pathobiology, School of Veterinary Medicine and Biomedical Sciences, Department of Veterinary Integrative Biosciences, Texas A&M University; 5 Department of Biology, Texas A&M University; 6 Caesar Kleberg Wildlife Research Institute, Department of Rangeland and Wildlife Sciences, Texas A&M University-Kingsville; 7 Government Canyon State Natural Area, Texas Parks and Wildlife Department; Tsinghua University, CHINA

## Abstract

Microplastic pollution has become a global concern and understanding its impact on wildlife requires effective sampling techniques that quantify exposure. In particular, non-lethal sampling techniques are needed for passerines for which microplastic exposure is poorly understood. In this study, we evaluated whether non-lethal proventricular gastric lavage can provide a representative sample of total microplastic ingestion in passerine birds. We sampled Brown-headed Cowbirds (*Molothrus ater*) (n = 105) from Government Canyon State Natural Area in San Antonio, Texas, United States (US). We performed gastric lavage to recover microplastics from each bird, before euthanizing them and dissecting gastrointestinal tracts. We recovered microplastics from 99% of birds. Gastric lavage recovered an average of 50.4% of ingested microplastics although recovery rate was highly variable (range: 0–100%, coefficient of variation: 59.52%), indicating much uncertainty in estimating individual total microplastic loads from gastric lavage. Sampling date influenced microplastic loads and recovery rates, which may be due to untested microplastic-environment interactions or may be an artifact of sampling conditions. Recovery rate was unaffected by time of day, bird age, sex, or body condition, or microplastic shape. Overall, our findings suggest that gastric lavage provides highly variable estimates of total gastrointestinal microplastics, and may be more appropriate for studies of recently ingested microplastics, only, that should be contained within the proventriculus.

## Introduction

Since the 1950s, global plastic production has increased exponentially [1], with over 8 billion metric tons produced as of 2017, 79% of which has been released into the environment [2]. Plastic pollution is highly persistent in the environment because most plastic products have high structural and chemical stability, and thus are not biodegradable. Instead, larger plastics typically fragment into microplastics [3], here defined as plastics between 1 and 5 mm in diameter [4,5], including microplastic fibers. Microplastics purposely manufactured for use in industry or that are found in cosmetics further contribute to this pervasive environmental issue [6].

Microplastics are ubiquitous in the environment, where they pose a physical and toxicological threat to wildlife including birds. Birds may consume microplastics directly by mistaking them for food items or other dietary supplements (*e.g.*, grit in seed eating birds, chalk in parrots), or indirectly through trophic transfer from prey items [7–12]. Birds can also ingest microplastics incidentally through other environmental interactions, such as preening and diving or through the incorporation of anthropogenic debris into nesting material [13,14]. The potential impacts of microplastic exposure on avian health are wide-ranging [15]. Birds can be impacted physically through “plasticosis” (*i.e.,* fibrotic scarring of the gastrointestinal tract), disruption of digestion, cecal inflammation, dysbiosis of the gut microbiome, and the creation of a false sense of satiety [15–18]. Microplastics can also impact birds toxicologically by the chemical byproducts or residual chemicals associated with plastic production and/or by environmental contaminants adsorbed to microplastics [15].

**Fig 1:**
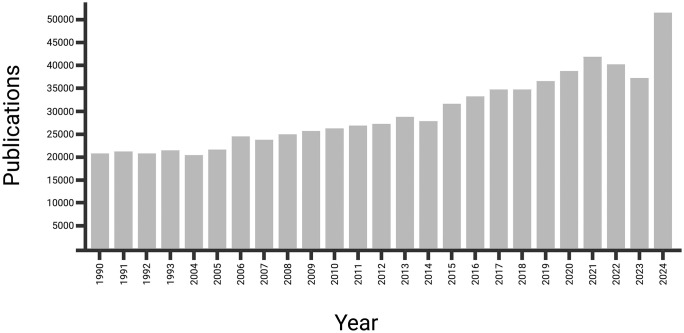
Publication results by year from January 1999 to December 2024 from a Web of Science records search. Search criteria included (keyword, title, abstract) using the terms “bird” or “avian” and “microplastic”. Note that Web of Science does not search all academic journals. Created with biorender.com.

Over the past 25 years, research related to birds and microplastics has been increasing at an accelerated pace (Fig 1). Microplastic exposure via ingestion has now been documented in many bird species, predominantly seabirds and other higher-trophic level birds (*i.e*., Accipitriformes, Strigiformes, and Falconiformes; [15]. However, lower-trophic and small-bodied birds, particularly passerines and near-passerines, have been largely overlooked [19]. Passerines are probably exposed to microplastics because of their highly diversified life-histories and cosmopolitan distributions, and the ubiquity of microplastics across ecosystems [3]. Additionally, there is strong evidence for trophic transfer of microplastics from invertebrates and low-trophic taxa to higher-trophic consumers, including passerines [20–23]. Given the precipitous decline in many passerine species over the last 50 years [24], studies that address microplastic exposure in these species are warranted.

Assessments of microplastic exposure in birds largely rely on salvaged carcasses, lethally sampled individuals, or opportunistic samples of feces and regurgitated stomach contents from live individuals [5]. Under scenarios where samples are collected from salvaged carcasses or rehabilitated wild birds, microplastic loads may not be representative of exposure at the population level if microplastic exposure increases the likelihood of significant health effects (*e.g.,* death, fatigue, excessive regurgitation; [5,25]. Thus, sampling of free-living wild birds may provide more realistic measures of exposure at the population level. Ideally, such sampling efforts should be non-lethal to allow for repeated sampling of the same individuals over time. Moreover, targeted non-lethal methods can increase the range of species and age classes studied [26]. Despite this need, there are few well-established methods for non-lethal microplastic sampling of free-living birds from species that do not regurgitate regularly upon capture or from species of small body sizes (*e.g.,* most passerines [5]).

Emetics (pharmaceutical agents that induce regurgitation) have been used for decades to sample stomach contents from small free-living birds, such as passerines and small seabirds, with varying success [26,27]. Some emetic compounds can be toxic to wildlife (e.g., apomorphine and tartar emetic), limiting their usefulness for field studies of wild birds. Others, such as syrup of ipecacuanha, are rarely toxic but dosages are critical to efficacy and toxicity, again limiting their usefulness for field studies, especially in understudied species [26]. Gastric lavage (*i.e.,* crop flushing or stomach flushing) has been suggested as a suitable non-lethal alternative for avian microplastic sampling and is used extensively for stomach content sampling in fish, reptiles, and amphibians [28,29]. In birds, it is often used to sample ingested plastics in seabirds (which naturally regurgitate to feed chicks) Hutton et al., 2008; Lavers et al., 2014), but gastric lavage has also been used to sample microplastics ingested by Clapper Rails (*Rallus creptians*) and Seaside Sparrows (*Ammospiza martima*), both species that do not naturally regurgitate to feed chicks, in coastal saltmarshes in Mississippi [30]. The efficacy of gastric lavage for microplastic sampling, however, has only been examined in seabirds, with mixed results. For example, in Northern Fulmars (*Fulmarus glacialis*) plastic quantities were lower in the proventriculus than the gizzard and gastric lavage recovered less plastics from the proventriculus than previously reported for fulmars via dissection [25,31]. However, in shearwaters (Procellariidae spp.), gastric lavage recovers between 78% and 94% of proventricular ingested microplastics in chicks as compared to dissection of carcasses [9,11]. In species that do not naturally regurgitate to feed chicks, the gastric isthmus inhibits regurgitation from the gizzard and lower gastrointestinal tract [25]. Thus, gastric lavage is assumed not to recover stomach contents past the proventriculus for these species [31]. A few major unanswered questions are (1) what proportion of total ingested microplastics is collected by gastric lavage in birds that do not naturally regurgitate to feed chicks, and (2) do lavaged microplastics correlate with total ingested microplastics [31]. While the proventriculus collects recently ingested plastics, health effects of plastics can occur at any point during digestion, with most health effects of nano- and microplastics being noted in intestinal tract [32], making total microplastics the more interesting measure for many studies and biomonitoring efforts. Passerine birds are the most common and widespread order of birds, and are widely used in ornithological study. Despite this, the efficacy of gastric lavage to recover microplastics has not been reported for birds that do not naturally regurgitate to feed chicks, including passerine birds, for which digestive physiology and regurgitation behavior can differ substantially from that of seabirds.

The aim of this study was to assess whether gastric lavage could be used as a non-lethal alternative to lethal sampling for estimation of total ingested microplastics in passerine birds. Specifically, we evaluated whether gastric lavage of the proventricular contents of Brown-headed Cowbirds (*Molothrus ater*) provided a representative sample of the total microplastic load in the gastrointestinal tract. We expected that microplastic quantities recovered by lavage would be lower than that of dissection due to the presence of the gastric isthmus, but that they would correlate with the total quantity of ingested microplastics. Under this scenario, we predicted that the proportion of total microplastics recovered by lavage would be similar across the population. However, we predicted that if, in fact, the proportion of total microplastics was not similar across the population, then characteristics of the individual bird and sample may mediate the recovery of microplastics, including sexes, age groups, body condition, time of capture, sampling date, or microplastic shape. We investigated these factors because sex (Brown-headed Cowbirds are strongly sexually dimorphic [33]) age, and body condition could influence foraging patterns and/or physiology in ways that may impact lavage efficacy (e.g., by affecting regurgitation potential). We used time of capture as an approximation for daily foraging patterns that may influence stomach fullness and/or digestive processes, since birds tend to forage more heavily in the morning and evening, even in captive settings [34]. These may affect the location of food items and microplastics in the gastrointestinal tract, and thus affect the recovery of microplastics in lavage samples. Lastly, microplastic shape could impact gastric lavage efficacy by influencing their motility. Microplastic fibers, for example, exhibit higher motility in laboratory settings and are more easily displaced by water [35,36], and thus may be more easily regurgitated. To evaluate these predictions, we combined gastric lavage in the field with subsequent euthanasia, dissection, and microplastic analysis.

## Materials and methods

### Study area

We sampled Brown-headed Cowbirds (hereafter cowbirds) trapped during a cowbird management program at Government Canyon State Natural Area [centered on 29.54941, −98.76478], an approximately 4,850-hectare natural wilderness in the Edwards Plateau Ecoregion, Texas, United States (US). The habitat is dominated by ashe juniper (*Juniperus ashei*), Texas persimmon (*Diospyros texana*), and live oak (*Quercus virginiana*) and is defined by limestone hills and plateaus. We sampled cowbirds from two trap locations, 2.9 km apart.

### Permitting and authorizations

All field sampling was performed under Texas Parks and Wildlife Department State Park Research Permit Number 13−23 and under Institutional Animal Care and Use Committee Approval (TAMU IACUC 2021−0042). Cowbirds were salvaged as a part of The Texas Parks and Wildlife Department cowbird management program and reported to the United States Fish and Wildlife Service in the annual reports for Government Canyon State Natural Area’s cowbird depredation program.

### Field methods

We trapped cowbirds using a ladder-trap baited with seed and a live decoy bird at each trap location between 15 March 2023 and 15 May 2023. Once captured, birds were housed in traps with *ad libitum* mixed wild bird seed and water until sampling. Sampling occurred on 4 April, 27 April, and 10 May of 2023. We removed birds in groups of 10–12 from each trap via hand capture or hand netting and assigned them a unique identifier. We performed two rounds of gastric lavage on each bird by injecting 15 mL of sterile deionized water through a stainless steel 6.3 cm curved, veterinary feeding tube for each round of lavage [30]. The lavaged samples were stored in a sterile liquid sample bag [37], labeled with the bird’s unique identifier. After gastric lavage, each cowbird was sedated via inhalation of 5% isoflurane suspended in paraffin oil and then euthanized via rapid cardiac compression [38]. We stored each carcass in Ziploc® bags labeled with the bird’s unique identifier and kept them on ice until returning to the laboratory at which point they were stored at −20°C pending analysis.

### Laboratory methods

We followed the best-practices outlined by Provencher et al. [5] to quantify and classify microplastics in our samples, defined as any plastic particle between 1 and 5 mm at its greatest dimension. While this size range follows established definitions of microplastics [4,15,39], it excludes particles smaller than 1 mm (*i.e.*, ultrafine microplastics and nanoplastics), which are often grouped within broader microplastic classifications. Thus, our estimates of total microplastic burden do not include smaller particles that may be a large part of the plastic burden for small passerines [40,41].

Each bird was prepared as a study skin or open wing and vouchered at the Biodiversity Research and Teaching Collections at Texas A&M, College Station, Texas, US (S1 Table), during which we identified the bird to age and sex [42]. We also took morphometric measurements, including mass (g, digital scale), flattened wing chord (mm, graduated wing bar), tail length (mm graduated wing bar), tarsus (mm, dial calipers), and exposed culmen length (mm, dial calipers). During this process, we dissected the entire gastrointestinal tract (*i.e.*, from the top of the esophagus to the cloaca) from each cowbird and wrapped them in lab-grade aluminum foil to avoid plastic contamination.

Gastrointestinal tract samples were digested following the procedures adapted from Provencher et al. [5]. Specifically, we washed each sample with deionized water into a sterile, 1-liter glass jar fitted with a metal lid, including any deionized water that was used to transfer the sample into the jar. We then performed alkaline digestion of samples using a solution of 1 mol/L (approximately 10%) potassium hydroxide and 10% Zep!® heavy-duty citrus terpene degreaser at 45°C in a VWR® biochemical oxygen demand low-temperature refrigerated incubator using a ratio of 1:2 sample to solution for 48–72 hours, or until large (*i.e*., greater than 5 mm) pieces of organic tissue were no longer present in the sample. Most plastic polymers are resistant to these conditions [43,44]. After digestion, samples were stored at −20°C to halt digestion or immediately filtered. For digested samples, we passed them through a 1–5 mm set of stacked sieves, where everything caught in the 1 mm sieve was considered a “microplastic candidate” even if it was slightly smaller than 1 mm. Lavage samples were not digested because they did not have large amounts of organic tissue [5]. Digested gastrointestinal samples and lavage samples (washed with deionized water) were filtered via vacuum filtration through a 57 mm Büchner funnel fitted with a labeled StonyLab® 15-micron quantitative silica-fiber filter paper. The filter paper was then removed from the Büchner funnel and placed into a covered petri dish, before being covered additionally with lab-grade aluminum foil to prevent airborne microplastic contamination, though we note that aluminum foil may introduce microplastic contaminants and blank sampling protocol should address this contamination [37]. Finally, microplastics in each sample were counted and classified to shape (*i.e.,* fiber, film, foam, fragment, or bead) via stereomicroscopy at 10-40x magnification. We used visual and physical methods to determine if particles were plastic following the methods of Shim et al. [45]. For example, to classify if particles were plastic we flame-heated a needle and brought it close to the particle. Microplastics were confirmed when the particle melted or permanently changed shape (S2 File). In contrast, non-plastic particles twisted, discolored, or remained unchanged [46].

For this study we were unable to perform more robust polymer analysis (*e.g.*, RAMAN spectroscopy, Fourier-transformed Infrared spectroscopy) and polymer identification due to budgetary constraints, and because this was outside the scope of our focal question. While visual and physical identification methods, including the hot-needle test, are widely used in microplastic studies [45], we acknowledge that they are subjective to the researcher and carry an inherent risk of misclassification, especially for microplastic particles < 500 μm [47]. However, in this study we restricted our size limits to 1–5 mm, which are more reliably identified as plastics via the hot-needle test [47]. Nevertheless, without confirmatory polymer identification (e.g., Fourier-Transformed Infrared Spectroscopy, Raman spectroscopy), our findings should be interpreted with this limitation in mind.

During laboratory work, all researchers wore an outer layer of natural fiber clothing, including a cotton lab coat and either latex or nitrile gloves to avoid contamination of samples via shedding of plastic-based synthetic fibers (primarily polyester, acrylic, nylon, and acetate). We did not perform laboratory analyses under a vacuum hood due to constraints of the laboratory layout but would recommend future studies perform analyses under a vacuum hood to reduce airborne contamination [37]. All deionized water and solutions used in analyses were filtered through a washed 300 μm mesh filter to remove any microplastics between 1–5 mm prior to use in analyses. Additionally, we prepared blank samples for all laboratory sessions to control for baseline contamination rates. Blank samples included controls during digestion and filtration steps, as well as filter papers exposed to airborne contamination at the same times as samples. We subtracted any microplastics found in the blanks from the total count of microplastics found in each sample processed on the same day.

### Statistical analyses

#### Representativeness of gastric lavage.

All statistical analyses were performed in R Statistical Software (v4.5.0) [48]. First, we calculated the total microplastic load for each individual by summing the number of microplastic particles recovered by gastric lavage and dissection. We considered this an approximation of what would be recovered by dissection of the gastrointestinal tract prior to gastric lavage. Recovery rate was estimated as the percentage of the total microplastic count that was recovered by lavage for each individual. To evaluate whether lavage was a representative sample of total microplastic load, we first calculated the mean, standard deviation, and coefficient of variation in recovery rates for all birds. Second, we constructed a linear regression model (normal error distribution, identity link function) in which total microplastic counts were predicted by lavaged microplastics counts.

We evaluated whether recovery rates were affected by sex, age, body condition, time of capture, or sampling date. First, we calculated Scaled Mass Index (*i.e.*, SMI – mass scaled to flattened wing chord length [49] as a proxy for individual body condition. SMI values were z-scored within sex to account for body size differences between male and female cowbirds [33]. Capture time for each bird was minutes after 10:00 AM (*i.e.*, when sampling commenced). Because we noted capture order for birds but not exact capture time, we estimated capture time in intervals of seven minutes (*i.e*., the approximate handling time per bird). We used success/trial syntax to create a quasibinomial regression model in the native stats package in R, fitted with a logit link function, with “successes” being represented by lavaged microplastic counts, “failures” being represented by the gastrointestinal microplastic count (*i.e.*, total microplastic count – lavaged microplastic count), and the number of trials represented by the total microplastic count. Recovery rate (i.e., “successes/trials) was predicted by sex (male, female), age (second-year, after hatch-year), body condition (estimated by SMI), approximate capture time, and date (as a categorical variable). Parameter estimates were derived from the global model. Lastly, for our quasibinomial regression model, we removed any samples for which we found no microplastics (n = 1), to avoid division by zero in our recovery rate calculations.

Lastly, to test if microplastic shape influenced recovery rate, we used generalized linear mixed models (beta error distribution). We constructed beta regression mixed models in the R package “glmmTMB” (v1.1.8; [50]) and hurdle transformed shape-specific recovery rates to constrain recovery rates to the interval (0,1). Shape-specific recovery rates were predicted by microplastic shape (i.e., fiber, fragment, film) and individual ID as a random effect, because individuals were in the dataset more than once if they had ingested multiple microplastic shapes.

#### Factors that may affect microplastic counts.

We also evaluated potential factors that may affect (1) total microplastic counts, (2) the count of lavaged microplastics, and (3) counts of microplastics in the gastrointestinal tract (*i.e.*, those not recovered in the lavage). For this, we constructed general linear models (GLMs; normal error distributions, identity link function) for each microplastic count metric separately with sex (male, female), age (second-year, after hatch-year), body condition (estimated by SMI), approximate capture time, and date (as a categorical variable) as explanatory variables. We used the “dredge()” function in the R package “MuMln” (v1.47.5; [51]) to calculate Akaike’s Information Criterion corrected for small sample size (AICc) and to rank candidate models. Models that were within 2 AICc units of the top-ranked model were considered informative and were model averaged (full) using the “model.avg()” function in the “MuMln” package according to the recommendations of Burnham and Anderson [52]. We calculated McFadden’s pseudo-R^2^ (R^2^_McF_) for each component model in our model set to demonstrate model likelihood and fitness. For our averaged models, we assessed likelihood and fitness of the model using the R^2^_McF_ of the component models, with the true R^2^_McF_ of the averaged model falling between the lowest and highest R^2^_McF_ values of the component models.

## Results

We found that some lavage samples (n = 15) analyzed on 22 May 2023 were contaminated with fibers that appeared to be from the cotton drawstring bags used in the field to contain birds prior to processing. Due to extensive contamination, we were unable to effectively test each fiber to confirm it as a microplastic and removed these samples from the final analyses. We also excluded individuals if they were euthanized before processing was completed, either in association with lavage stress/injury (n = 5) or an injury discovered during capture (n = 2). Our final dataset included 78 individuals for which we had sex, age, mass, flattened wing chord, and both lavage and gastrointestinal samples.

### Representativeness of lavage

**Fig 2:**
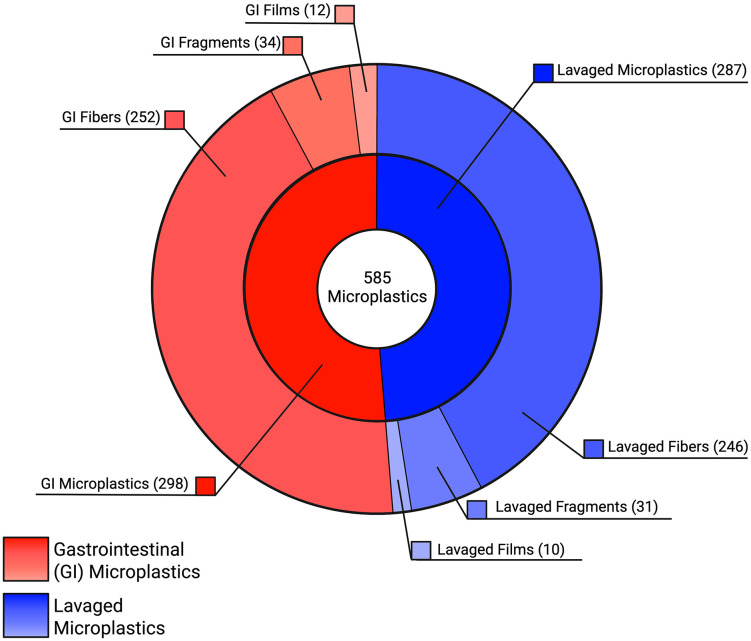
Multi-level pie chart demonstrating the proportion of microplastics found in gastric lavage and GI samples. Blue sections represent microplastics recovered from lavage and red sections represent microplastics detected in the gastrointestinal tracts in cowbirds, broken down by microplastic shape. Created with biorender.com.

**Fig 3:**
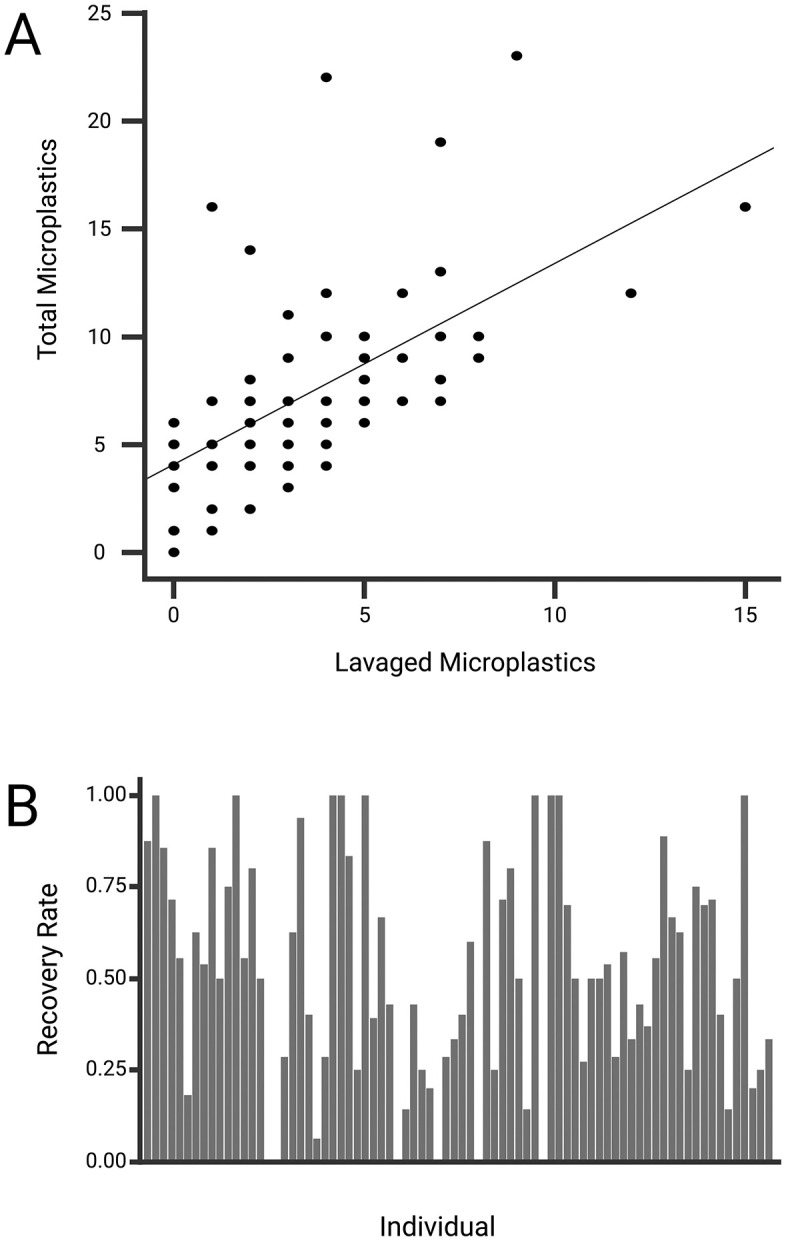
Visual comparison of microplastic counts in lavage and gastrointestinal tract samples of cowbirds. **(A)** Relationship between the total number of microplastics recovered from lavage samples and the number of microplastics present in dissected gastrointestinal tracts of the same individuals. Each point represents one bird; the solid line shows the best-fit linear regression between the two measures. **(B)** Individual recovery rates for lavage samples, calculated as the proportion of microplastics recovered relative to the total count per bird.

We found microplastics in 77 of the 78 (98.71%) birds included in our dataset. There were a total of 585 microplastic pieces amongst birds with an average of 7.5 microplastics per bird, (range: 0–23; Table 1). A total of 287 microplastic pieces were found in lavage samples and 298 in gastrointestinal samples. Fibers made up the majority of the microplastics identified in both lavage and gastrointestinal samples, followed by fragments, and then films in nearly equal amounts for both lavage and gastrointestinal samples (Fig 2). We did not find any beads or foams. The percentage of total microplastics recovered by gastric lavage (*i.e*., “recovery rate”) for each bird was highly variable and ranged from 0% to 100%, with a mean of 50.47% (Wald CI = ± 11.27%; coefficient of variation: 59.52%; SD = 30.04%; Fig 3). Additionally, linear regression revealed a moderately strong, positive, linear association between lavage and total microplastic counts (p < 0.001, t = 6.660, 95% CI = 0.396 ± 0.0.059, R^2^ = 0.3602; Fig 3).

**Table 1:** Table of summary statistics for the total ingested microplastics found in our samples (n = 78) by microplastic shape.

*Microplastic*	*Minimum*	*Maximum*	*Mean*	*Standard Deviation*	*95% CI*
*All*	*0*	*23*	*7.50*	*4.34*	*7.50 *∓* 0.98*
*Fibers*	*0*	*20*	*6.38*	*3.66*	*6.38 *∓* 0.83*
*Fragments*	*0*	*14*	*0.83*	*1.75*	*0.83 *∓* 0.40*
*Films*	*0*	*3*	*0.28*	*0.64*	*0.28 *∓* 0.15*

“CI” stands for confidence interval.

Recovery rates were unaffected by sex, age, body condition (*i.e.,* SMI), or time of capture. Date of capture did affect recovery rate and rates were lower on 10 May than on 4 April (p = 0.008) (Table 2). Recovery rates were also unaffected by microplastic shape (films: p = 0.559, z = −0.584, 95% CI = −0.215 ± 0.368; fragments: p = 0.129, z = 1.516, 95% CI = 0.396 ± 0.261; fibers: p = 0.559, z = 0.584, 95% CI = 0.215 ± 0.368).

**Table 2:** Table of results for a Quasibinomial Regression Model for recovery rate predicted by the interaction between sex and age, time of day estimated by minutes since 10:00 AM, the within-sex z-score of scaled mass index (SMI), and sampling date.

Model	Factor	Estimate	Standard Error	z-value	p-value	R^2^_McF_
Recovery Rate ~ Sex * Age + Time + SMI + Date						0.20
	(Intercept)	−0.01	0.44	−0.03	0.975	
	Sex (M)	−0.19	0.33	−0.58	0.565	
	Age (SY)	0.39	0.48	0.81	0.424	
	Time	0.00	0.00	1.18	0.24	
	SMI	−0.05	0.15	−0.33	0.74	
	Date					
	27 April	−0.52	0.32	−1.62	0.11	
	10 May	−1.00	0.37	−2.71	0.01*	
	Sex:Age	0.33	0.57	0.58	0.56	

“M” refers to male individuals, and “SY” refers to second-year birds. R^2^_McF_ is McFadden’s pseudo-R^2^. Asterisks denote significant p-values (p < 0.05). Our reference level for the date is 04 April, which is represented by the intercept.

### Factors that may affect microplastic counts

Total microplastic counts, lavaged microplastic counts, and gastrointestinal microplastic counts were each predicted by several models within 2 AICc units of the top-ranked model (Table 3). Thus, for each candidate set, we estimated parameters via model averaging those models for which ΔAICc < 2. Model averaged estimates suggested that there were no important predictors of total microplastic counts, but there were important effects of date on lavaged and gastrointestinal microplastic counts (Table 4). Counts of lavaged microplastics were lower on 10 May than on 4 April (p = 0.008) and counts of microplastics in the gastrointestinal samples were higher on 27 April than on 4 April (p = 0.030) (Table 4).

**Table 3:** Results of AICc model selection, including all models with ΔAICc < 2 and the null model.

Global Model	Candidate Model	K	AICc	ΔAICc	AICc Weight	Cumulative AICc Weight	Log Likelihood	R^2^_McF_
Total Microplastic Count ~ Sex * Age + SMI^a^ + Date + Time								
	*Total Microplastic Count ~ Day*							
		4	452.95	0.00	0.20	0.20	−222.20	0.06
	*Total Microplastic Count ~ Day + Sex*							
		5	453.52	0.57	0.15	0.35	−221.34	0.08
	*(null) Total Microplastic Count ~ 1*							
		2	453.56	0.61	0.15	0.50	−224.70	<0.001
	*Total Microplastic Count ~ Day + SMI*							
		5	453.69	0.74	0.14	0.64	−221.43	0.08
	*Total Microplastic Count ~ Day + Sex + SMI*							
		6	454.21	1.26	0.11	0.75	−220.52	0.10
	*Total Microplastic Count ~ Sex*							
		3	454.48	1.52	0.09	0.84	−224.08	0.02
	*Total Microplastic Count ~ SMI*							
		3	454.71	1.75	0.08	0.92	−224.19	0.01
	*Total Microplastic Count ~ Age + Day*							
		5	454.90	1.95	0.08	1.00	−222.03	0.07
Lavaged Microplastic Count ~ Sex * Age + SMI + Date + Time								
	*Lavaged Microplastic Count ~ Age + Day*							
		5	379.46	0.00	0.30	0.30	−184.31	0.17
	*Lavaged Microplastic Count ~ Age + Day + SMI*							
		6	380.33	0.88	0.19	0.49	−183.58	0.18
	*Lavaged Microplastic Count ~ Age + Day + Sex*							
		6	380.92	1.47	0.14	0.64	−183.87	0.17
	*Lavaged Microplastic Count ~ Age + Day + Time*							
		6	381.24	1.78	0.12	0.76	−184.03	0.17
	*Lavaged Microplastic Count ~ Day + SMI*							
		5	381.35	1.90	0.12	0.88	−185.26	0.14
	*Lavaged Microplastic Count ~ Age + Day + Time + SMI*							
		7	381.42	1.96	0.11	0.99	−182.91	0.19
	*(null) Lavaged Microplastic Count ~ 1*							
		2	386.83	7.37	0.01	1.00	−191.33	<0.001
GI Microplastic Count ~ Sex * Age + SMI + Date + Time								
	*GI Microplastic Count ~ Date*							
		4	417.02	0.00	0.28	0.28	−204.23	0.07
	*GI Microplastic Count ~ Age*							
		3	417.85	0.84	0.18	0.46	−205.76	0.03
	*(null) GI Microplastic Count ~ 1*							
		2	417.95	0.93	0.17	0.63	−206.89	<0.001
	*GI Microplastic Count ~ Date + Age*							
		5	418.24	1.22	0.15	0.78	−203.70	0.08
	*GI Microplastic Count ~ Date + Sex*							
		5	418.83	1.81	0.11	0.89	−204.00	0.07
	*GI Microplastic Count ~ Time*							
		3	418.94	1.93	0.11	1.00	−206.31	0.02


^a^SMI is a proxy for body condition, represented by the scaled mass index z-scored within sex. Higher SMI scores correlate to higher body condition within sex.

K is the number of parameters in the candidate model. AICc is Akaike’s Information Criterion corrected for small sample sizes. AICc weight is the relative likelihood of the model, where 1.0 is most likely. Cumulative AICc weight is the sum of the relative likelihoods for each model and all higher ranked models. Log Likelihood is an estimate of the goodness-of-fit for each model, with larger magnitudes being more fit. R^2^_McF_ is McFadden’s pseudo-R^2^.

**Table 4:** Results of model averaging using the full-averaged (non-conditional) models.

Averaged Model	Factor	Estimate	Standard Error	Adj. Standard Error	z-value	p-value
*Total Microplastic Count*						
	(Intercept)	6.93	1.09	1.10	6.30	<0.001*
	Date					
	27 April	1.75	1.23	1.25	1.40	0.16
	10 May	−0.86	1.32	1.34	0.64	0.52
	Sex (M)	1.31	1.05	1.07	1.22	0.22
	SMI	−0.58	0.49	0.50	1.15	0.25
	Age (SY)	0.60	1.06	1.08	0.56	0.58
*Lavaged Microplastic Count*						
	(Intercept)	3.81	0.87	0.88	4.31	<0.01*
	Age (SY)	1.33	0.68	0.69	1.94	0.05
	Date					
	27 April	−0.22	0.77	0.78	0.28	0.78
	10 May	−2.15	0.80	0.81	2.65	0.01*
	SMI	−0.45	0.33	0.33	1.35	0.18
	Sex (M)	0.61	0.67	0.68	0.90	0.37
	Time	0.00	0.00	0.00	0.88	0.38
*GI Microplastic Count*						
	(Intercept)	3.35	1.10	1.11	3.03	<0.01*
	Date					
	27 April	2.11	0.96	0.97	2.17	0.03*
	10 May	1.24	1.01	1.03	1.21	0.23
	Age (SY)	−1.06	0.85	0.87	1.22	0.22
	Sex (M)	0.57	0.86	0.87	0.66	0.51
	Time	0.00	0.00	0.00	1.06	0.29

Models include total microplastic counts, lavaged microplastic counts, and gastrointestinal (GI) microplastic counts predicted by the interaction between sex and age, time of day estimated by minutes since 10:00 AM, the within-sex z-score of scaled mass index (SMI), and sampling date. “M” refers to male individuals, and “SY” refers to second-year birds. Asterisks denote significant p-values (p < 0.05).

Lastly, seven of the 105 individuals for which we performed lavage (6.67%) needed to be euthanized prior to fully collecting lavage samples. Two of these (1.90%) were previously injured in the trap, and five (4.76%) were euthanized due to severe symptoms of morbidity during lavage (*i.e.*, limpness, heavy breathing, glassy eyes).

## Discussion

Our results provide mixed support for gastric lavage as a representative sample of total gastrointestinal microplastics in a passerine bird. The high variation in recovery rates between birds suggests that gastric lavage is an imprecise proxy for total gastrointestinal microplastics in the 1–5 mm size range. However, lavaged microplastic quantities were predictive of total 1–5 mm microplastic quantities in the gastrointestinal tract, indicating that while variation exists at the individual level, at the population level gastric lavage may be a representative sample of microplastic quantities for passerine birds. Moreover, recovery rate was largely unaffected by demographic or sampling variables (*i.e.,* sex, age, body condition, time of capture), suggesting that lavage does not bias toward any of these demographic groups or sampling conditions. Recovery rate was affected by sampling date with samples collected on the 4 April having a higher recovery rate than those collected on 10 May 2023. This could be due to procedural differences including familiarity with methods by the field crew. However, we observed a decline in recovery rate across time, which is not consistent with a procedural learning curve (Table 2). Future studies are needed to determine if environmental and/or procedural factors are responsible for differences in recovery rate between sampling dates.

In birds, the gastric isthmus typically restricts lavage to sampling the proventricular contents [9]. Thus, our results indicate that proventricular microplastic quantities probably have low correlation to microplastic quantities in the lower intestinal tract, although future research is needed to confirm this. Similar to prior studies [9,31], we found lower recovery of microplastics by lavage than by dissection. Interestingly, Hutton et al. [9] found that shearwaters exhibited lower microplastic recovery by lavage even when only proventricular contents were examined, suggesting that lavage does not fully extract microplastics from the proventriculus. Together with our findings, this suggests that the recovery of microplastics by gastric lavage may vary not only due to differences between microplastic quantities in the proventriculus versus remaining gastrointestinal tract, but also due to differences in the completeness of regurgitation. We expected that daily eating patterns could affect both the location of microplastics in the gastrointestinal tract and the completeness of regurgitation. To evaluate and account for this we included time of day in our analysis and this was not an important predictor of recovery rate. A constant diet of mixed wild bird seed was provided *ad libitum* in the trap, which may have affected eating patterns. However, birds in both wild and captive settings generally forage more heavily in the morning and evening [34], and we assumed similar patterns for the cowbirds in this study.

To our knowledge, this is the first assessment of the variation in lavage recovery rate for individuals of any bird species. Prior verifications of gastric lavage for microplastic sampling compared microplastic quantities recovered by gastric lavage and by dissection in independent groups of seabirds [9,31], and thus could not assess individual variation in recovery rates. For shearwaters, [9] estimated that 76–94% of ingested microplastics in the proventriculus were recovered by lavage. Our Wald 95% confidence interval in Brown-headed Cowbirds suggests that lavage recovers 39–62% of total ingested microplastics in passerines. This large difference in recovery estimates is probably mostly explained by differences in sampling and our microplastic size limits (*i.e.,* 1–5 mm) being more restrictive than other studies. Our recovery rate estimates reflect total gastrointestinal microplastics, and not just proventricular microplastics, while those of Hutton et al. [9] reflect proventricular contents, only. Differences in species biology may also contribute to the lower recovery rates observed in our study. *Molothrus* cowbirds are obligate brood parasites and do not raise their own offspring or form pellets to remove indigestible matter [33]. Thus, regurgitation is not a common behavior for cowbirds, as it is for most seabirds. The microplastic quantities we observed in cowbirds were also relatively small compared to those found in seabirds [11], and it is possible that larger microplastic quantities are more easily recovered by lavage (although we found no indication of this in our analyses).

We found no evidence that microplastic counts by lavage or dissection, or recovery rates were affected by bird sex, age, body condition, or approximate capture time. We also found no evidence that microplastic shape or total microplastic load influenced recovery rate. Thus, birds with higher or lower microplastic loads were no more likely to regurgitate more or less microplastics. The only factor that influenced microplastic quantities or recovery rate was the date of sampling. Relatively little is known about the temporal and environmental conditions that may affect microplastic detection in the environment – especially terrestrial environments – due, in part, to the high variability of detection methods and non-standard definitions of “microplastics” [53]. The impact of date on microplastic counts and recovery rate could be an artifact of these unknown environmental-microplastics interactions or differences in human or bird behavior by date.

### Caveats and limitations

Our aim was to determine whether gastric lavage resulted in a representative sample of microplastics in the entire gastrointestinal tract. However, because our analyses excluded particles <1 mm, the recovery rates reported here reflect only the larger microplastic fraction. Smaller microplastic particles (<1 mm) are increasingly recognized as a dominant fraction ingested by many bird species and are an emerging concern for terrestrial birds and small passerines [40,41]. Smaller particles may differ in their retention, movement within the gastrointestinal tract, and likelihood of regurgitation during lavage, which could affect recovery positively or negatively if smaller particles are more or less mobile. Our assessment of gastric lavage recovery rates applies only to the 1–5 mm fraction, and not to total plastic ingestion across all size classes.

Additionally, we did not perform polymer confirmation (e.g., FTIR or Raman spectroscopy), potentially leading to some misclassification of microplastic particles as non-plastic and *vice versa*. We do not expect a large degree of misclassification because our physical identification methods aligned with accepted coarse identification protocols [45] and misidentification by visual identification is predominantly a problem for plastics smaller than 1 mm [47] that were not included in our study. However, any misclassification could alter our estimates of total microplastic burden, and this may affect specific shapes or polymers more than others.

As mentioned previously, gastric lavage probably only recovers plastics from the proventriculus [31], and we did not separately dissect the proventriculus from the rest of the gastrointestinal tract. Thus, we were unable to also evaluate the efficacy of gastric lavage for removal of proventricular microplastics in passerine birds. For shearwaters, gastric lavage removes 76–94% of proventricular microplastics [9], and the efficacy may be similar for passerine birds. We also used passive trapping techniques for cowbirds. This meant that we were unable to precisely control the duration of captivity for each bird, or to assess differences in foraging patterns as birds adjusted to captivity, which may affect the location or quantities of ingested microplastics in the gastrointestinal tract. We recommend that future studies investigate these relationships between food ingestion, stress, and lavage recovery rates.

Our field observations of sample contamination and morbidity in lavage samples highlight the need for careful procedures and training. Firstly, we used standard cotton drawstring bags to hold birds after capture and prior to sampling. Fifteen of our samples were removed from our analyses because contamination with cotton fibers from these bags was indistinguishable from microplastic fibers. Avoiding direct contact of birds and samples with synthetic fibers in the field would likely correct for this contamination in future studies. Secondly, the deleterious impacts of gastric lavage on birds are species-specific [5]. Our study had a mortality rate of 4.76% associated with gastric lavage, which is higher than the reported 0.3% to 2.3% in multiple studies of seabirds [5,54], but less than the 8.0% mortality rate for chemical emetics in a similarly sized seabird, Leach’s Storm-Petrel (*Oceanodroma leucorhoa*) [26]. Thus, we recommend that species-specific reactions, including mortality and recovery rates, should be assessed and considered before choosing lavage as a sampling technique.

### Recommendations for future research

We recommend the following actions be taken for future studies on microplastic ingestion in wild birds:

1. Future studies should prioritize standardizing and verifying non-lethal methods to sample ingested microplastics, including gastric lavage, in a variety of species, because the efficacy of these methods is probably affected by gastrointestinal physiology.
2. Gastric lavage may not result in a representative sample of ingested microplastics in the entire gastrointestinal tract. Thus, it may not be an appropriate sampling method for microplastics in passerine birds when researchers are interested in fine-scale differences in total ingested microplastics between individuals. When differences are expected to be large, or researchers are interested only in proventricular contents, gastric lavage may be appropriate.
3. Plastic size and polymer type might affect retention, movement within the gastrointestinal tract, and potential for regurgitation during lavage. Future studies should consider quantifying the full range of microplastic sizes in potential samples, particularly particles smaller than 1 mm including nanoplastics and performing spectroscopic polymer identification to reduce uncertainty and improve comparability between studies.
4. For studies estimating total microplastic exposure from gastric lavage counts, researchers should account for the variability in gastric lavage as a sampling method, particularly when performing power analyses and calculating estimated sample sizes. For example, we calculated the minimum sample size needed in future studies to estimate recovery rates under high variance for cowbirds, assuming recovery rate is fairly normally distributed, estimating the margin of error as the 8% reported for shearwaters [9,11], and estimating the population recovery rate with the mean recovery rate in our study (50.47%). Using the sample size formula for population proportions, *n* = *Z* ^*2*^ (*p*)(1-*p*)/*E* ^*2*^, where *n* is the estimated sample size, *Z* is the z-score at the 95% confidence level, *p* is the sample proportion, and *E* is the desired margin of error, future studies in cowbirds or passerines of similar exposure-levels should have a minimum sample size of at least 149 individuals within each population of interest to approximate the population recovery rate within 8% of the true population recovery rate. However, if future studies choose to include polymer identity as a predictor variable, researchers should ensure sample sizes are sufficient to test for effects of polymer type and potential interactions with other particle traits, such as shape, without inflating unexplained variation or risking overinterpretation.
5. Because sampling date appears to affect recovery rate and lavaged microplastic counts, considering this effect in future study designs will contribute to a more comprehensive assessment of microplastic exposure across bird populations.

## Conclusions

Our findings indicate that non-lethal gastric lavage does not result in a consistently representative sample of total ingested microplastics within the 1–5 mm size range in a passerine bird. However, microplastic quantities obtained by gastric lavage in our study did predict total microplastic quantities within the 1–5 mm size range, and were not influenced by bird sex, age, or body condition, or time of sampling, suggesting that gastric lavage may be useful for population-level studies of total ingested microplastics, or where the interest is in recently ingested microplastic particles (although very small plastic particles may reach the gizzard very quickly; [31]). Current methods to assess the status of microplastic contamination in the environment are highly heterogeneous [55] both in field and laboratory settings [55,56], and often only focus on aquatic systems. This has resulted in a significant gap in our understanding of microplastic contamination in terrestrial organisms [57], including a lack of bioindicators for microplastics in the non-aquatic systems [56]. Furthermore, smaller-bodied and lower-trophic species such as passerines are highly diversified and widespread globally, making them potential sentinels for environmental microplastic contamination. Non-lethal methods are an important tool for studies using birds as sentinels for microplastic contamination in the environment [5], which requires targeted sampling, and for rigorous studies of microplastic exposure in birds of conservation concern.

## Supporting information

S1 Table Table of TCWC numbers, specimen type, and special numbers for each vouchered specimen. TCWC Numbers are the unique identifier assigned to each bird upon installation into the Biodiversity Research and Teaching Collections, formerly known as the Texas Cooperative Wildlife Collections (TCWC). These numbers should be the reference numbers for any loan requests for these specimens. Prep numbers are unique to the preparator and are sequential to the number of specimens added by the associated preparator. If the preparator is labeled as “2023”, the specimen was prepared by a student volunteer or intern.
(DOCX)

S2 File Video of a black microplastic fiber melting when a flame-heated needle is brought close to it. (MP4)
