# Supplementary material for: Gastric lavage may not be representative of total microplastic ingestion for a wild passerine bird
Source: PLoS One. 2025 Oct 23;20(10):e0334891. doi: 10.1371/journal.pone.0334891 (PMC12548855; doi:10.1371/journal.pone.0334891)
Supplement: S1 Table — TCWC Numbers are the unique identifier assigned to each bird upon installation into the Biodiversity Research and Teaching Collections, formerly known as the Texas Cooperative Wildlife Collections (TCWC). These numbers should be the reference numbers for any loan requests for these specimens. Prep numbers are unique to the preparator and are sequential to the number of specimens added by the associated preparator. If the preparator is labeled as “2023”, the specimen was prepared by a student volunteer or intern. (DOCX) [file pone.0334891.s001.docx]

| **TCWC Number** | **Species** | **Date** | **Locality** | **Sex** | **Preparator** | **Prep No** | **Study ID** |
| --- | --- | --- | --- | --- | --- | --- | --- |
| 30337 | *Molothrus ater* | 04 April 2023 | Government Canyon SNA | M | Prestridge, H.L. | 2394 | GCB012 |
| 30338 | *Molothrus ater* | 04 April 2023 | Government Canyon SNA | M | Prestridge, H.L. | 2395 | GCB026 |
| 30353 | *Molothrus ater* | 04 April 2023 | Government Canyon SNA | M | 2023 | 183 | GCA009 |
| 30354 | *Molothrus ater* | 04 April 2023 | Government Canyon SNA | M | 2023 | 184 | GCA007 |
| 30355 | *Molothrus ater* | 04 April 2023 | Government Canyon SNA | M | 2023 | 185 | GCB020 |
| 30356 | *Molothrus ater* | 04 April 2023 | Government Canyon SNA | F | 2023 | 186 | GCA013 |
| 30357 | *Molothrus ater* | 04 April 2023 | Government Canyon SNA | M | 2023 | 187 | GCSNA001 |
| 30358 | *Molothrus ater* | 04 April 2023 | Government Canyon SNA | M | 2023 | 188 | GCA3 |
| 30359 | *Molothrus ater* | 04 April 2023 | Government Canyon SNA | F | 2023 | 189 | GCA5 |
| 30360 | *Molothrus ater* | 04 April 2023 | Government Canyon SNA | F | 2023 | 190 | GCB019 |
| 30361 | *Molothrus ater* | 04 April 2023 | Government Canyon SNA | M | 2023 | 191 | GCA004 |
| 30362 | *Molothrus ater* | 04 April 2023 | Government Canyon SNA | M | 2023 | 192 | GCA002 |
| 30363 | *Molothrus ater* | 04 April 2023 | Government Canyon SNA | M | 2023 | 193 | GCB023 |
| 30364 | *Molothrus ater* | 04 April 2023 | Government Canyon SNA | M | 2023 | 194 | GCA015 |
| 30365 | *Molothrus ater* | 04 April 2023 | Government Canyon SNA | M | 2023 | 195 | GCB018 |
| 30366 | *Molothrus ater* | 04 April 2023 | Government Canyon SNA | M | 2023 | 196 | GCB027 |
| 30367 | *Molothrus ater* | 04 April 2023 | Government Canyon SNA | M | 2023 | 197 | GCB017 |
| 30368 | *Molothrus ater* | 04 April 2023 | Government Canyon SNA | M | 2023 | 198 | GCB006 |
| 30369 | *Molothrus ater* | 04 April 2023 | Government Canyon SNA | M | 2023 | 199 | GCB024 |
| 30370 | *Molothrus ater* | 04 April 2023 | Government Canyon SNA | M | 2023 | 200 | GCB021 |
| 30371 | *Molothrus ater* | 04 April 2023 | Government Canyon SNA | M | 2023 | 201 | GCA014 |
| 30372 | *Molothrus ater* | 04 April 2023 | Government Canyon SNA | M | 2023 | 202 | N/A |
| 30398 | *Molothrus ater* | 27 April 2023 | Government Canyon SNA | F | 2023 | 228 | GCA043 |
| 30399 | *Molothrus ater* | 27 April 2023 | Government Canyon SNA | M | 2023 | 229 | GCA031 |
| 30400 | *Molothrus ater* | 27 April 2023 | Government Canyon SNA | F | 2023 | 230 | GCA050 |
| 30401 | *Molothrus ater* | 27 April 2023 | Government Canyon SNA | F | 2023 | 231 | GCB055 |
| 30402 | *Molothrus ater* | 27 April 2023 | Government Canyon SNA | M | 2023 | 232 | GCA038 |
| 30403 | *Molothrus ater* | 27 April 2023 | Government Canyon SNA | F | 2023 | 233 | GCA039 |
| 30404 | *Molothrus ater* | 27 April 2023 | Government Canyon SNA | F | 2023 | 234 | GCA035 |
| 30405 | *Molothrus ater* | 27 April 2023 | Government Canyon SNA | M | 2023 | 235 | GCB068 |
| 30406 | *Molothrus ater* | 27 April 2023 | Government Canyon SNA | F | 2023 | 236 | GCA044 |
| 30407 | *Molothrus ater* | 27 April 2023 | Government Canyon SNA | M | 2023 | 237 | GCB066 |
| 30408 | *Molothrus ater* | 27 April 2023 | Government Canyon SNA | M | 2023 | 238 | GCA042 |
| 30409 | *Molothrus ater* | 27 April 2023 | Government Canyon SNA | M | 2023 | 239 | GCB075 |
| 30410 | *Molothrus ater* | 27 April 2023 | Government Canyon SNA | F | 2023 | 240 | GCB058 |
| 30411 | *Molothrus ater* | 27 April 2023 | Government Canyon SNA | M | 2023 | 241 | GCA033 |
| 30412 | *Molothrus ater* | 27 April 2023 | Government Canyon SNA | F | 2023 | 242 | GCA032 |
| 30413 | *Molothrus ater* | 27 April 2023 | Government Canyon SNA | F | 2023 | 243 | GCA030 |
| 30414 | *Molothrus ater* | 27 April 2023 | Government Canyon SNA | F | 2023 | 244 | GCA037 |
| 30415 | *Molothrus ater* | 27 April 2023 | Government Canyon SNA | F | 2023 | 245 | GCA036 |
| 30416 | *Molothrus ater* | 27 April 2023 | Government Canyon SNA | M | 2023 | 246 | GCB071 |
| 30417 | *Molothrus ater* | 27 April 2023 | Government Canyon SNA | M | 2023 | 247 | GCB053 |
| 30418 | *Molothrus ater* | 27 April 2023 | Government Canyon SNA | M | 2023 | 248 | GCA049 |
| 30419 | *Molothrus ater* | 27 April 2023 | Government Canyon SNA | F | 2023 | 249 | GCB060 |
| 30420 | *Molothrus ater* | 27 April 2023 | Government Canyon SNA | F | 2023 | 250 | GCA045 |
| 30421 | *Molothrus ater* | 27 April 2023 | Government Canyon SNA | F | 2023 | 251 | GCB056 |
| 30422 | *Molothrus ater* | 27 April 2023 | Government Canyon SNA | F | 2023 | 252 | GCB070 |
| 30423 | *Molothrus ater* | 27 April 2023 | Government Canyon SNA | M | 2023 | 253 | GCB072 |
| 30424 | *Molothrus ater* | 27 April 2023 | Government Canyon SNA | F | 2023 | 254 | GCA041 |
| 30425 | *Molothrus ater* | 27 April 2023 | Government Canyon SNA | M | 2023 | 255 | GCB057 |
| 30426 | *Molothrus ater* | 27 April 2023 | Government Canyon SNA | F | 2023 | 256 | GCB073 |
| 30427 | *Molothrus ater* | 27 April 2023 | Government Canyon SNA | M | 2023 | 257 | GCB059 |
| 30428 | *Molothrus ater* | 27 April 2023 | Government Canyon SNA | F | 2023 | 258 | GCA034 |
| 30429 | *Molothrus ater* | 27 April 2023 | Government Canyon SNA | F | 2023 | 259 | GCA029 |
| 30430 | *Molothrus ater* | 27 April 2023 | Government Canyon SNA | F | 2023 | 260 | GCA040 |
| 30431 | *Molothrus ater* | 27 April 2023 | Government Canyon SNA | F | 2023 | 261 | GCB065 |
| 30432 | *Molothrus ater* | 27 April 2023 | Government Canyon SNA | F | 2023 | 262 | GCA052 |
| 30433 | *Molothrus ater* | 27 April 2023 | Government Canyon SNA | F | 2023 | 263 | GCB074 |
| 30434 | *Molothrus ater* | 27 April 2023 | Government Canyon SNA | F | 2023 | 264 | GCA048 |
| 30435 | *Molothrus ater* | 27 April 2023 | Government Canyon SNA | M | 2023 | 265 | GCB062 |
| 30436 | *Molothrus ater* | 27 April 2023 | Government Canyon SNA | M | 2023 | 266 | GCB061 |
| 30437 | *Molothrus ater* | 27 April 2023 | Government Canyon SNA | M | 2023 | 267 | GCB076 |
| 30438 | *Molothrus ater* | 27 April 2023 | Government Canyon SNA | F | 2023 | 268 | GCA047 |
| 30439 | *Molothrus ater* | 27 April 2023 | Government Canyon SNA | M | 2023 | 269 | GCB054 |
| 30440 | *Molothrus ater* | 27 April 2023 | Government Canyon SNA | F | 2023 | 270 | GCA028 |
| 30442 | *Molothrus ater* | 10 May 2023 | Government Canyon SNA | F | 2023 | 272 | GCA093 |
| 30443 | *Molothrus ater* | 10 May 2023 | Government Canyon SNA | M | 2023 | 273 | GCB097 |
| 30444 | *Molothrus ater* | 10 May 2023 | Government Canyon SNA | M | 2023 | 274 | GCB105 |
| 30445 | *Molothrus ater* | 10 May 2023 | Government Canyon SNA | F | 2023 | 275 | GCB101 |
| 30446 | *Molothrus ater* | 10 May 2023 | Government Canyon SNA | F | 2023 | 276 | GCB104 |
| 30447 | *Molothrus ater* | 10 May 2023 | Government Canyon SNA | M | 2023 | 277 | GCA091 |
| 30448 | *Molothrus ater* | 10 May 2023 | Government Canyon SNA | M | 2023 | 278 | GCB099 |
| 30449 | *Molothrus ater* | 10 May 2023 | Government Canyon SNA | F | 2023 | 279 | GCB087 |
| 30450 | *Molothrus ater* | 10 May 2023 | Government Canyon SNA | F | 2023 | 280 | GCB102 |
| 30451 | *Molothrus ater* | 10 May 2023 | Government Canyon SNA | F | 2023 | 281 | GCA082 |
| 30452 | *Molothrus ater* | 10 May 2023 | Government Canyon SNA | M | 2023 | 282 | GCA089 |
| 30453 | *Molothrus ater* | 10 May 2023 | Government Canyon SNA | M | 2023 | 283 | GCA090 |
| 30454 | *Molothrus ater* | 10 May 2023 | Government Canyon SNA | F | 2023 | 284 | GCA078 |
| 30455 | *Molothrus ater* | 10 May 2023 | Government Canyon SNA | F | 2023 | 285 | GCA088 |
| 30456 | *Molothrus ater* | 10 May 2023 | Government Canyon SNA | F | 2023 | 286 | GCA096 |
| 30457 | *Molothrus ater* | 10 May 2023 | Government Canyon SNA | F | 2023 | 287 | GCA080 |
| 30458 | *Molothrus ater* | 10 May 2023 | Government Canyon SNA | M | 2023 | 288 | GCB095 |
| 30459 | *Molothrus ater* | 10 May 2023 | Government Canyon SNA | F | 2023 | 289 | GCA083 |
| 30460 | *Molothrus ater* | 10 May 2023 | Government Canyon SNA | M | 2023 | 290 | GCB103 |
| 30461 | *Molothrus ater* | 10 May 2023 | Government Canyon SNA | F | 2023 | 291 | GCB094 |
| 30462 | *Molothrus ater* | 10 May 2023 | Government Canyon SNA | F | 2023 | 292 | GCB098 |
| 30463 | *Molothrus ater* | 10 May 2023 | Government Canyon SNA | M | 2023 | 293 | GCA081 |
| 30464 | *Molothrus ater* | 10 May 2023 | Government Canyon SNA | F | 2023 | 294 | GCA077 |
| 30465 | *Molothrus ater* | 10 May 2023 | Government Canyon SNA | F | 2023 | 295 | GCA084 |
| 30466 | *Molothrus ater* | 10 May 2023 | Government Canyon SNA | F | 2023 | 296 | GCA086 |
| 30467 | *Molothrus ater* | 10 May 2023 | Government Canyon SNA | M | 2023 | 297 | GCA085 |
| 30468 | *Molothrus ater* | 10 May 2023 | Government Canyon SNA | F | 2023 | 298 | GCA092 |
| 30584 | *Molothrus ater* | 04 April 2023 | Government Canyon SNA | M | Andringa, R.K | 558 | GCA008 |
| 30585 | *Molothrus ater* | 04 April 2023 | Government Canyon SNA | M | Andringa, R.K | 559 | GCA011 |
| 30586 | *Molothrus ater* | 04 April 2023 | Government Canyon SNA | F | Andringa, R.K | 560 | GCA010 |
| 30587 | *Molothrus ater* | 04 April 2023 | Government Canyon SNA | M | Andringa, R.K | 561 | GCB025 |
| 30588 | *Molothrus ater* | 04 April 2023 | Government Canyon SNA | M | Andringa, R.K | 562 | GCA016 |
| 30598 | *Molothrus ater* | 27 April 2023 | Government Canyon SNA | M | Andringa, R.K | 572 | GCB067 |
| 30610 | *Molothrus ater* | 27 April 2023 | Government Canyon SNA | M | Andringa, R.K | 584 | GCB069 |
| 30611 | *Molothrus ater* | 27 April 2023 | Government Canyon SNA | F | Andringa, R.K | 585 | GCA031 |
| 30663 | *Molothrus ater* | 27 April 2023 | Government Canyon SNA | M | Prestridge, H.L. | 2410 | GCB 064 |
| 30664 | *Molothrus ater* | 27 April 2023 | Government Canyon SNA | M | Prestridge, H.L. | 2411 | GCB 063 |
| 30665 | *Molothrus ater* | 27 April 2023 | Government Canyon SNA | M | Prestridge, H.L. | 2412 | GCB 046 |
| 30673 | *Molothrus ater* | 10 May 2023 | Government Canyon SNA | M | Prestridge, H.L. | 2420 | GCA 079 |
| 30674 | *Molothrus ater* | 10 May 2023 | Government Canyon SNA | M | Prestridge, H.L. | 2421 | GCB 100 |
